# Three new species of spiny throated reed frogs (Anura: Hyperoliidae) from evergreen forests of Tanzania

**DOI:** 10.1186/s13104-015-1050-y

**Published:** 2015-04-25

**Authors:** Simon P Loader, Lucinda P Lawson, Daniel M Portik, Michele Menegon

**Affiliations:** Department of Environmental Sciences, University of Basel, Biogeography Research Group, Basel, 4056 Switzerland; Committee on Evolutionary Biology, University of Chicago, 1025 E. 57th Street, Culver Hall 402, Chicago, IL 60637 USA; Field Museum of Natural History, 1400 S. Lake Shore Drive, Chicago, IL 60605 USA; University of Cincinnati, 614 Rieveschl Hall, Cincinnati, OH 45221 USA; Museum of Vertebrate Zoology and Department of Integrative Biology, University of California, 3101 Valley Life Sciences Building, Berkeley, California 94720 USA; Tropical Biodiversity Section, Science Museo of Trento, Via della Scienza e del lavoro, 38122 Trento, Italy

## Abstract

**Background:**

The East African spiny-throated reed frog complex (*Hyperolius spinigularis*, *H. tanneri*, and *H. minutissimus*) is comprised of morphologically similar species with highly fragmented populations across the Eastern Afromontane Region. Recent genetic evidence has supported the distinctiveness of populations suggesting a number of cryptic species. We analyse newly collected morphological data and evaluate the taxonomic distinctiveness of populations.

**Results:**

We find three new distinct species on the basis of morphological and molecular evidence. The primary morphological traits distinguishing species within the *Hyperolius spinigularis* complex include the proportions and degree of spinosity of the gular flap in males and snout-urostyle length in females. Other features allow the three species to be distinguished from each other (genetics). We refine the understanding of *H. minutissimus* which can be found in both forest and grassland habitats of the Udzungwa Mountains, and provide more details on the call of this species. Further details on ecology are noted for all species where known.

**Conclusions:**

Three new species are described and we narrow the definition and distribution of *Hyperolius spinigularis* and *H. minutissimus* in East Africa. The spiny-throated reed frogs have highly restricted distributions across the fragmented mountains of the Eastern Afromontane region. Given the newly defined and substantially narrower distributions of these spiny-throated reed frog species, conservation concerns are outlined.

**Electronic supplementary material:**

The online version of this article (doi:10.1186/s13104-015-1050-y) contains supplementary material, which is available to authorized users.

## Background

The East African spiny-throated reed frog complex (*Hyperolius spinigularis*, *H. tanneri*, and *H. minutissimus*) is comprised of morphologically similar species occupying isolated mountaintops across the Eastern Afromontane region. Lawson [1] provided molecular evidence supporting the recognition of these three taxa and their distinctiveness from one another. The validity of these species has never been seriously questioned, though suggestions of more than one species in *Hyperolius spinigularis* and *H. minutissimus* have been remarked upon in the literature [1,2].

*Hyperolius spinigularis* was described by Stevens [3] based on material collected from Mulanje in Malawi. Subsequently, Schiøtz [2] reported the presence of this species *ca*. 1300 km north in East Usambara Mountains in Tanzania, though questioned its taxonomic placement. Schiøtz [2] noted (p. 166) “it is questionable whether the two forms should be separated subspecifically.” Schiøtz expanded on morphological differences between the populations stating “the males are of the same size, the females seem smaller in the northern sample. The breadth of the protective flap is greater than the length and often weakly bilobed in the type material, circular or almost circular in the northern sample” [2], p. 166). This initial observation of geographically distinct morphological variation cast doubt on whether the Malawi and northern Tanzanian populations were part of the same lineage or better regarded as distinct species.

Since its initial description, *Hyperolius spinigularis* has been documented to occur across a much larger range in East Africa beyond Malawi and East Usambara. This includes Udzungwa [4], Nguru [5] and Uluguru [1]. However, the record provided by Schiøtz and Westergaard [4] of *H. spinigularis* in the forests of the Udzungwa was incorrect. The Udzungwa species record is *Hyperolius minutissimus* – a species Schiøtz and Westergaard [4] described*. H. minutissimus* is found across forest and grassland sites in the Udzungwa. Tellingly, Schiøtz and Westergaard [4] (p. 8) noted about their Udzungwa forest material: “A chirping voice, acoustically similar to that of *H. minutissimus*, was noted from specimens kept in plastic bags.” Also noting on their collection (p. 8), “The new material seems in these characters closest to the southern population; the gular flap is slightly heart-shaped with width greater than length in most specimens” which is also in line with the broader proportions of the gular flap in *H. minutissimus*. Beyond the Udzungwas, Portik et al. [6] reported *H.* cf. *spinigularis* from the Namuli massif in Mozambique, and Lawson [1] detailed the occurrence of *H. spinigularis* from the Uluguru in Tanzania. Overall the range of species referable to *H. spinigularis* has been recorded from fragmented and distant locations across the elements of the Afromontane region of East Africa. The species *H. minutissimus* was described by Schiøtz [2] 10 km West from Njombe in the vicinity of the Southern Highlands with populations also recorded further north from the grasslands [4] and forests [1] of the Udzungwa.

Lawson [1] outlined considerable genetic diversity in the species *H. minutissimus* and *H. spinigularis* above the species level. This included samples from recent surveys in Tanzania in the Rubeho and Livingstone Mountains and in Mozambique (Namuli) by the authors of this paper. Analyses by Lawson et al. [7] provided further evidence of these divergences, which sampled across known populations and extended the geographic scope of these analyses.

These recently discovered genetically divergent lineages also prove to be diagnosable using morphological characters, and here we provide formal species descriptions and a revised identification key for these lineages. We reassess the geographic distributions of the spiny-throated reed frogs based on these discoveries – clarifying previous uncertainty in the distribution of species, and address the conservation implications based on the newly defined and narrowed ranges.

## Methods

### Molecular data and analysis

Specimens collected from our fieldwork were fixed in either 95% ethanol or 5% formalin, and subsequently stored in 70% ethanol. Samples of muscle and/or liver were taken from representative individuals and preserved in 95% ethanol, these specimens are listed in Additional file 1. Lawson [1] and Lawson et al. [7] provide details on the approaches and genes used in this study, which include one mitochondrial gene and three nuclear loci (mitochondrial: NADH dehydrogenase subunit 2; nuclear: POMC, C-myc and Rag-1). In Lawson et al’s [7] publication their Table 1 provide details on samples included in this study, including their origin and associated Genbank numbers. Phylogenetic relationships were estimated between all individuals using likelihood and Bayesian methods, including BEAST, RAxML, and BPP [8-10], using data from Lawson et al. [7]. Species trees were constructed based on these trees and by using species delimitation methods in *BEAST and BPP [10,11]. To examine species boundaries across the reconstructed phylogeny we applied three species delimitation methods: a Bayesian implementation of the General Mixed Yule-Coalescent model (“bGMYC” package v. 1.0.2 for R, [12]) using trees from the BEAST analysis, a Bayes Factor species Delimitation (BFD;[13]) to compare alternative scenarios for the *H. minutissimus, H. spingularis,* and *H. tanneri* species complex using alternate *BEAST species trees (See Additional file 1: Table S1), and a joint estimation of the species tree and species delimitation in BPP3 [10]. Each of the coalescent species tree analyses were run twice.

**Table 1:** **Descriptive statistics for the three new species for 17 morphological characters**

**Males**																		
**Species**		**SUL**	**HW**	**HLD**	**HLDDJ**	**NS**	**IN**	**EN**	**EE**	**IO**	**TL**	**THL**	**TFL**	**FL**	**FLL**	**HL**	**WGF**	**HGF**
*H. burgessi (19)*																		
	Average	18.5	6.4	5.8	6.5	1.0	1.9	1.9	3.8	2.4	9.2	8.8	5.9	7.7	4.4	5.0	5.0	5.1
	St. Dev	1.3	0.4	0.4	0.5	0.1	0.1	0.1	0.5	0.2	0.5	0.5	0.3	0.5	0.4	0.2	0.4	0.4
	Range	16.4-20.3	5.4-7.3	5.2-6.5	5.9-7.4	0.9-1.2	1.6-2.1	1.7-2.3	2.6-5	2.2-3	8.4-9.8	8-9.9	5.1-6.3	6.7-8.6	3.9-5.8	4.5-5.5	4.2-6.1	4.3-6.1
*H. davenporti (10)*																		
	Average	18.9	6.5	5.4	6.4	1.0	1.9	1.9	3.8	2.4	9.1	9.1	5.8	7.4	4.4	5.4	5.4	5.0
	St. Dev	1.0	0.3	0.3	0.4	0.1	0.1	0.1	0.2	0.2	0.4	0.4	0.4	0.7	0.2	0.3	0.3	0.4
	Range	17.3-20.2	6-7.1	5-6.1	5.6-6.9	0.9-1.2	1.8-2.1	1.8-2	3.4-4	2.1-2.7	8.4-9.8	8.4-9.7	5-6.4	6.2-8.5	4.1-4.7	5.1-5.8	4.6-5.8	4.4-5.6
*H. minutissimus (13)*																		
	Average	20.3	6.8	6.1	6.5	1.0	2.0	2.0	4.2	2.4	9.9	9.5	6.2	8.8	4.7	5.8	5.9	4.5
	St. Dev	1.2	0.5	0.4	0.0	0.1	0.1	0.1	0.4	0.2	0.6	0.6	0.5	0.7	0.4	0.4	0.4	0.4
	Range	18.8-22.7	6-7.5	5.5-6.8	6.5-6.5	0.9-1.1	1.7-2.1	1.7-2.3	3.4-4.7	1.9-2.8	8.7-11	8.6-10.7	5.5-7.3	7.8-10.3	4-5.5	5-6.8	5.2-6.9	3.7-5.1
*H. spinigularis (9)*																		
	Average	19.6	6.9	6.1	6.9	1.1	1.9	2.0	3.9	2.5	9.9	8.8	6.2	8.2	4.5	5.3	5.1	4.0
	St. Dev	1.0	0.4	0.4	0.4	0.1	0.1	0.2	0.2	0.3	0.3	0.6	0.4	0.7	0.2	0.4	0.4	0.3
	Range	18.6-21.8	6.2-7.6	5.8-7.1	6.5-7.8	1-1.2	1.7-2.1	1.7-2.2	3.7-4.3	2-2.9	9.3-10.2	7.6-9.6	5.6-6.9	7.6-9.6	4.2-5	4.7-5.9	4.3-5.5	3.6-4.4
*H. tanneri (2)*																		
	Average	22.2	6.9	6.1	6.9	1.2	2.0	2.1	4.1	2.7	11.1	8.9	6.0	9.5	4.7	6.3	NA	NA
	St. Dev	0.4	0.9	0.6	0.5	0.1	0.2	0.3	0.8	0.4	0.7	1.6	1.1	1.1	0.4	0.7	NA	NA
	Range	21.9-22.5	6.2-7.5	5.7-6.5	6.5-7.2	1.2-1.1	1.8-2.1	2.3-1.9	3.5-4.7	2.4-3	11.6-10.6	7.7-10	5.2-6.7	8.7-10.3	4.4-5	5.8-6.8	NA	NA
**Females**																		
*H. burgessi (28)*																		
	Average	24.3	8.5	7.2	8.3	1.3	2.4	2.3	4.8	3.1	12.1	11.2	7.6	10.3	5.4	7.1		
	St. Dev	1.2	0.5	0.6	0.7	0.1	0.1	0.3	0.3	0.3	0.8	0.9	0.4	0.7	0.3	0.4		
	Range	21.3-25.9	7.6-9.6	6.1-8.3	6.5-9.4	1-1.5	2.1-2.6	1.7-2.8	4-5.3	2.3-3.8	10.6-13.1	9.8-12.9	6.9-8.4	9-11.7	4.6-5.9	6-7.8		
*H. davenporti (2)*																		
	Average	27.0	9.2	6.9	8.4	1.3	2.3	2.6	5.2	2.8	12.9	13.0	8.6	10.7	6.3	7.4		
	St. Dev	0.1	0.0	0.0	0.3	0.1	0.1	0.1	0.2	0.1	0.4	0.6	0.1	0.3	0.7	0.2		
	Range	26.9-27	9.2-9.2	6.9-6.9	8.2-8.6	1.2-1.3	2.2-2.3	2.5-2.7	5-5.3	2.7-2.9	12.6-13.1	12.5-13.4	8.5-8.6	10.5-10.9	5.8-6.8	7.2-7.5		
*H. ukwiva (2)*																		
	Average	28.7	10.0	8.0	9.8	1.5	2.8	2.7	5.5	3.2	14.2	14.1	8.9	12.7	7.5	9.0		
	St. Dev	0.9	0.8	0.1	0.7	0.2	0.4	0.1	0.2	0.1	1.1	0.8	0.2	0.5	0.4	0.8		
	Range	28-29.3	9.4-10.5	7.9-8.1	9.3-10.3	1.3-1.6	2.5-3	2.6-2.8	5.3-5.6	3.1-3.3	13.4-15	13.5-14.6	8.7-9	12.3-13	7.2-7.7	8.4-9.6		
*H. spinigularis (3)*																		
	Average	24.5	8.7	7.3	8.8	1.3	2.3	2.6	4.9	3.1	12.9	11.8	8.2	11.1	5.6	7.0		
	St. Dev	1.1	0.6	0.5	0.6	0.0	0.1	0.1	0.1	0.3	0.3	0.6	0.5	0.8	0.4	0.4		
	Range	23.6-25.7	8.3-9.4	6.9-7.8	8.3-9.5	1.3-1.3	2.3-2.4	2.5-2.7	4.8-5	2.8-3.4	12.7-13.3	11.4-12.5	7.7-8.6	10.2-11.8	5.3-6	6.6-7.3		
*H. tanneri (2)*																		
	Average	26.7	9.2	7.6	8.7	1.5	2.4	2.7	5.2	2.9	15.0	11.2	7.7	10.7	6.1	7.4		
	St. Dev	4.3	1.5	1.1	0.7	0.3	0.5	0.2	0.5	0.4	5.7	3.3	2.3	3.0	1.7	1.5		
	Range	23.6-29.7	8.1-10.2	6.8-8.4	8.2-9.2	1.3-1.7	2-2.7	2.5-2.8	4.8-5.5	2.6-3.2	10.9-19	8.8-13.5	6.1-9.3	8.5-12.8	4.9-7.3	6.3-8.4		

### Morphology

Material was examined in the following institutions: The Natural History Museum, London (BMNH); Field Museum, Chicago (FMNH); California Academy of Sciences, San Francisco (CAS); Museo Tridentino di Scienze Naturali, Trento (MTSN), and University of Dar es Salaam (UDSM) (see in Additional file 1: Table S1). Morphological measurements were taken using dial callipers, to the nearest 0.1 mm using Mitutoyo Absolute Digimatic Calipers (CD-6”C) with the aid of a Leica MZ8 stereo microscope (Leica Microsystems GmbH, Wetzlar, Germany). Only fully-grown specimens (adult color pattern and adult size) were measured. Sex was determined by the presence or absence of gular flap in adult specimens. Measurements in this analysis were length: Snout-Urostyle Length (SUL), Head Width (HW), Head Length Diagonal from corner of mouth (HLD), Head Length Diagonal from jawbone end (HLDJ), Nostril-Snout (NS), Inter-narial (IN), Eye to Nostril (EN), Eye Distance (EE), Inter-orbital (IO), Tibiafibula Length (TL), Thigh Length (THL), Tibiale Fibulare Length (TFL), Foot Length (FL), Forelimb Length (FLL), Hand Length (HL), Width of Gular Flap (WGF) Height of Gular Flap (HGF). Furthermore, qualitative characters were investigated: gular shape, proportions, and spinosity to assess species differences. In order to assess the morphometric distinctness of these species we also conducted Principal Component analyses on log-transformed data using various packages in R [14-16]. See Lawson et al. [7] for further explanation of these results.

### Acoustic information

Advertisement calls were recorded with an Olympus LS-10 PCM digital stereo audio recorder equipped with a Sony directional microphone. The calls were analyzed using the software package Raven 1.2 [17].

## Results and Discussion

### Phylogeny and species delimitation

**Figure 1:**
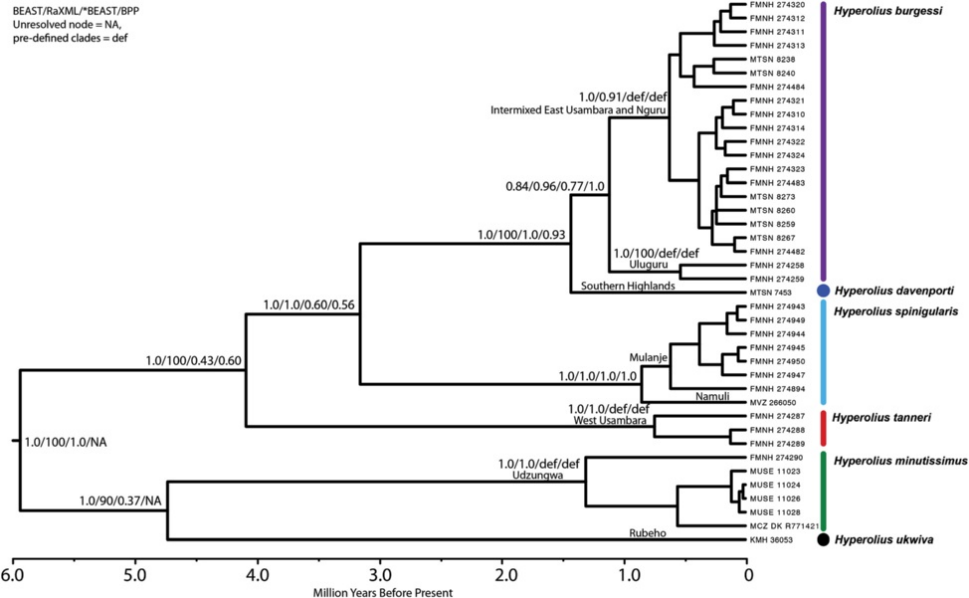
Bayesian maximum clade credibility chronogram of the spiny-throated reed frog species complex. Support for clades is shown on branches as well as location of clades.

All methods agreed on the optimal resolution of the evolutionary relationships within the clade of spiny-throated frogs, which consists of a well-supported monophyletic assemblage (Figure 1). *Hyperolius minutissimus* and *H. ukwiva* sp. nov. form a group which is a sister group to *H. davenporti* sp. nov., *H. burgessi* sp. nov., *H. tanneri*, and *H. spinigularis*. Within the latter clade it is shown that *H. tanneri* is well supported as the sister species to *H. davenporti* sp. nov., *H. burgessi* sp. nov., and *H. spinigularis. Hyperolius davenporti* sp. nov. and *H. burgessi* sp. nov. form a close grouping. Species delimitation approaches agreed with the taxonomic units recognized and clades recovered in the phylogeny (see Systematics section below: Additional file 1: Table S1) (see also Lawson, et al. [7].

### Morphology

**Figure 2:**
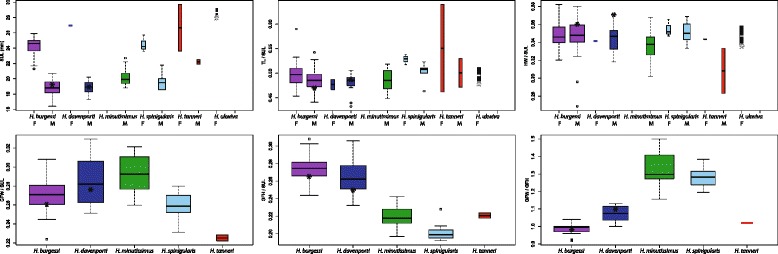
Plots of Snout – Urostyle Length (SUL), TL/SUL, HW/SUL, GFW/SUL, GFH/SUL, and GFW/GFH. Plots are grouped according to species and sex where appropriate, and color-coded following Figures 1 and 5. Boxes represent the interquartiles. Whiskers extend to the most extreme data point no more than 1.5 times the interquartile range from the box. Width of each box reflects relative sample sizes (square-roots of the number of observations). Asterix mark the holotype measurement.

Measurements for specimens are given in Additional file 2. Summary statistics of each species and characters are given in Table 1. Many traits distinguish species including SUL (M/F), TL/SUL (M/F), HW/SUL (M), GFH/SUL (M), GFW/GFH (F) (Figure 2, Table 2). PCA analysis of males shows largely overlapping results, though *H. burgessi* sp. nov. and *H. minutissimus* are distinct (see Lawson et al. [7]). PC1 is evenly representative of all traits. PCA analysis of females shows large areas of overlap, though the two *H. ukwiva* sp. nov. and two *H. tanneri* individuals are outside of the centroids of overlap for other species. PC1 represents 62% of variance and is even across traits with strongest influence from head width, snout-urostyle length, and leg measurements (see Supplementary Data Figure S3 and Table three in Lawson et al. [7]). Discriminant function analysis of males showed complete segregation between species, and females were distinguished for all species except for *H. spinigularis* and *H. burgessi* sp. nov., which were entirely overlapping (Supplementary Table S3, Lawson et al. [7]). Differentiation in males is largely driven by height of the gular flap (HGF). In females, differentiation is strongly dominated by tibia length and snout-urostyle length.

**Table 2:** **AMOVA tables for each morphometric variable/ratio for species with sex as a covariate**

**Females**					
SUL	Df	Sum Sq	Mean Sq	F value	Pr (>F)
Species	4	0.079	0.020	5.973	0.001
Residuals	32	0.105	0.003		
TL/SUL	Df	Sum Sq	Mean Sq	F value	Pr (>F)
Species	4	0.023	0.006	0.323	0.861
Residuals	32	0.570	0.018		
HW/SUL	Df	Sum Sq	Mean Sq	F value	Pr (>F)
Species	4	0.002	0.001	0.286	0.885
Residuals	32	0.065	0.002		
**Males**					
SUL	Df	Sum Sq	Mean Sq	F value	Pr (>F)
Species	4	0.116	0.029	7.941	0.000
Residuals	48	0.176	0.004		
TL/SUL	Df	Sum Sq	Mean Sq	F value	Pr (>F)
Species	4	0.013	0.003	1.641	0.179
Residuals	48	0.094	0.002		
HW/SUL	Df	Sum Sq	Mean Sq	F value	Pr (>F)
Species	4	0.037	0.009	1.962	0.115
Residuals	48	0.224	0.005		
GFW/SUL	Df	Sum Sq	Mean Sq	F value	Pr (>F)
Species	4	0.126	0.032	5.736	0.001
Residuals	47	0.258	0.005		
GFH/SUL	Df	Sum Sq	Mean Sq	F value	Pr (>F)
Species	4	0.816	0.204	51.880	0.000
Residuals	47	0.185	0.004		
GFW/GFH	Df	Sum Sq	Mean Sq	F value	Pr (>F)
Species	4	0.905	0.226	84.290	0.000
Residuals	47	0.126	0.003		

Df = Degrees of Freedom. Sq = squares. F value = F statistic. Pr (>F) = probability of F test (*p* value).

### Systematics

#### *Hyperolius burgessi* sp. nov.

##### Holotype

**Figure 3:**
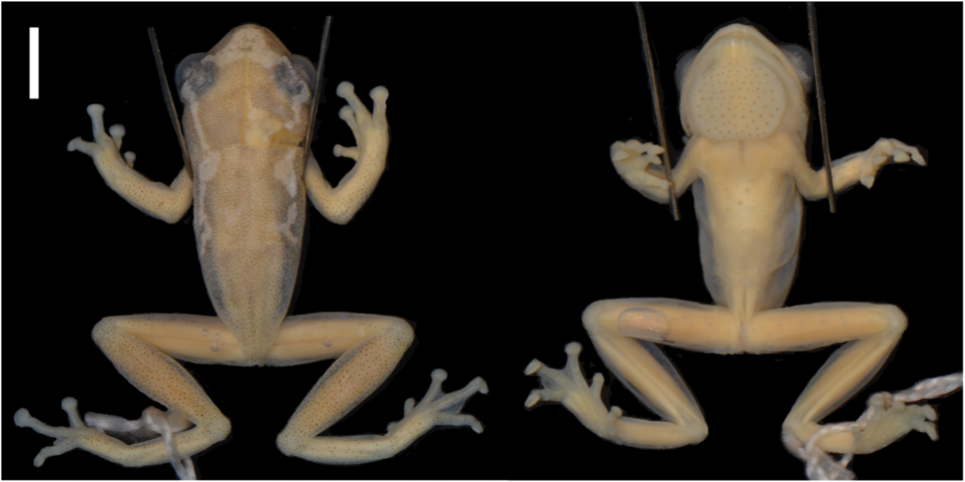
Dorsal and ventral views of the holotype of *H. burgessi* BMNH 1974.299. Bar = 5 mm.

BMNH 1974.299 (male) collected in Amani Nature Reserve, East Usambara Mountains, Tanzania (−5.2 S, 38.6167 E) by Alice Grandison (Figure 3).

##### Paratypes

We restrict paratype material to localities within the East Usambara on the basis that further detailed morphological/molecular analysis might uncover additional cryptic lineages. *Males*: BMNH 1974.295-298, BMNH 1974.300-303, BMNH 1974.304, same collection data as holotype; CAS 169977, CAS 169979–169980, CAS 169982–169985, CAS 169987–169990, CAS 169992–169994, CAS 169997–169998, CAS 170000–170005 collected in East Usambara Mountains, Tanzania by Robert Drewes and Jens Vindum, April 1988; FM 274310–274312, FM 274322 collected in East Usambara Mountains, Tanzania by Lucinda Lawson. *Females*: CAS 169258–169262, CAS 169945–169946, CAS 169976, CAS 169981, CAS 169991, CAS 169995–169996 collected in various localities in East Usambara Mountains, Tanzania by Robert Drewes and Jens Vindum, April 1988; FM 274313–274314, FM 274320–274321, FM 274323–274324 collected in in various localities in East Usambara Mountains, Tanzania by Lucinda Lawson.

##### Referred material

*Males*: MTSN 8238, MTSN 8240–8241, MTSN 8247, MTSN 8259–8260, MTSN 8266–8267 collected in Pemba, Nguru Mountains, Tanzania by Michele Menegon; FM 274259–60 collected in Uluguru Nature Reserve, Uluguru North, Uluguru Mountains, Tanzania by Lucinda Lawson. *Females*: MTSN 8265, MTSN 8271, MTSN 8273, MTSN 8278 collected in Nguru Mountains, Tanzania by Michele Menegon; FM 274258 collected in Uluguru Nature Reserve, Uluguru North, Uluguru Mountains, Tanzania by Lucinda Lawson.

##### Diagnosis

**Figure 4:**
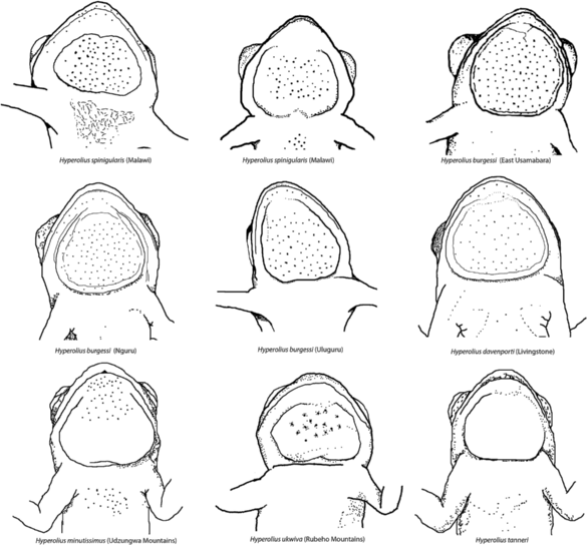
Schematic drawings of the ventral view of head region of spiny throated complex group.

**Figure 5:**
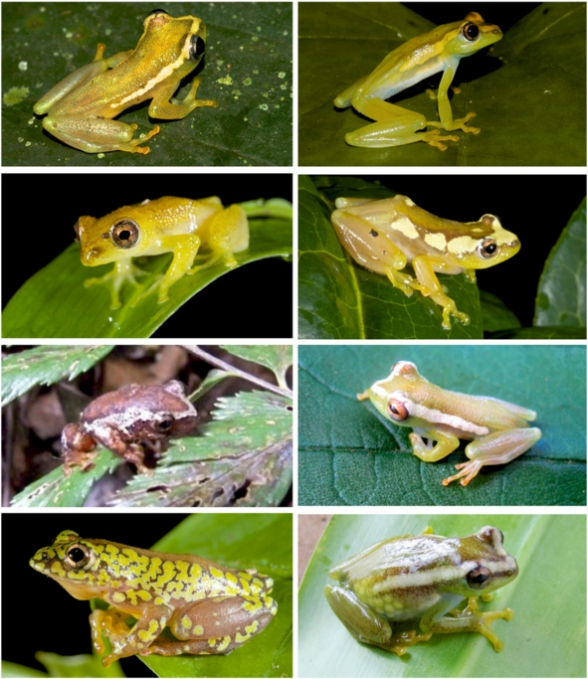
Elevational map and distribution of the six spiny-throated species in East Africa. Colour codes for species are: red = *H. tanneri,* purple = *H. burgessi*, green = *H. minutissimus*, dark blue = *H. davenporti*, light blue = *H. spinigularis*, and black *= H. ukwiva*. Mountain blocks containing spiny-throated species are referenced.

Horizontal pupil with distinctive gular flap in males. As with most other members of the clade of spiny-throated frogs (*Hyperolius spinigularis, H. davenporti, H. minutissimus*), *H. burgessi* also has the presence of dermal asperities (including the body and chin region) on the ventrum, unique amongst hyperoliids. The presence of asperites on the gular flap diagnoses this species from *H. tanneri,* for which they are absent. The even distribution of dermal asperites on the gular flap differs from the anteriorly positioned distribution of asperites in *H. minutissimus* and *H. ukviwa*. Furthermore, in males, the species has a distinctive gular flap morphology which differs from other members of the genus. *H. burgessi* males have a rounded gular flap which is not bilobed - distinctive from *H. spinigularis*. The shape of the gular flap also narrows anteriorly, being much smaller in size to the base of the gular flap, which is different from the gular flap of *H. davenporti* that is more equal in size anteriorly and posteriorly. Furthermore, the shape of the gular flap in males is different than *H. davenporti* in usually having an equal or greater height than width (see Figures 2, 3 and 4; Tables 1 and 2). Based on molecular comparisons the species is genetically distinct from close relatives (*H. burgessi*, *H. spinigularus*, and *H. tanneri*,), and is minimally 2% pairwise divergent from its closest relative based on mtDNA (ND2. Table 3; see Figure 1). *Hyperolius burgessi* also has a largely allopatric distribution with respect to other species in the complex (Figure 5).

**Table 3:** **Average nucleotide divergences in mitochondrial data (ND2) between species and populations**

	**1**	**2**	**3**	**4**	**5**	**6**
1. *H. burgessi* (East Usambara + Nguru)						
2. *H. burgessi* (Uluguru)	1.7%					
3. *H. tanneri*	6.4%	6.5%				
4. *H. minutissimus*	9.0%	9.1%	8.3%			
5. *H. spinigularis*	7.1%	6.6%	5.8%	7.9%		
6. *H. ukwiva*	12.5%	11.9%	10.8%	7.0%	10.9%	
7. *H. davenporti*	2.0%	2.3%	5.7%	9.0%	5.8%	11.3%

##### Description of holotype

Small to moderate sized hyperoliid. Pupil horizontal. Snout blunt slightly rounded. Canthus rostralis angular, being slightly convex on the horizontal plane and slightly concave on the vertical plane. Distance between eyes is 4.1 mm and the inter orbital distance is 2.4 mm. The inter-narial distance is 2.0 mm, greater than narial distance to the eye (1.8 mm). The nostril to snout is 1.0 mm. The width of head (6.9 mm) equaling 0.36 length of body (19.2 mm). The gular flap width is less than (5.0 mm) the height (5.1 mm). The gular flap is rounded, thickened and not bilobed, anteriorly narrowing so that overall shape is wide based hexagon. It is marked by black asperities (*ca*. 80) evenly distributed across the whole of the gular flap. Some asperities, sparsely distributed, though more concentrated, on the apex of the chin (mentum). Tibio-tarsal articulation of the adpressed hind limb reaching the eye. Tibio-tarsal (9.1 mm) is almost equal to thigh length (8.5 mm). The tibiable fibulare length is 5.9 mm. The toes have expanded fleshy discs with the foot being 7.8 mm. Webbing is extensive reaching the base of the fleshy discs on all toes apart from the first toe where it only reaches the first tubercle. The forelimb length is 4.2 mm, less than the hand length (5.0 mm). The hands have expanded, rounded fleshy discs. Webbing just reaching distal subarticular tubercle of the outer finger, reaching distal subarticular tubercle of the 4th toe on both sides. Dorsal skin surface granular with a single minute black asperites surmounting many of the granules. Ventral skin surface strongly granular with black asperities restricted to the mentum, gular disc, abdomen and undersurfaces of the femur. Ventral asperities much more prominent than those of the dorsum.

##### Paratypes

Head and body proportions in close agreement with those of the holotype (Figure 2, Table 1). The distribution of the asperities of the males are in close agreement with that of the holotype. The proportions of the gular flap, diagnostic for the species, shows some variation which means care needs to be taken in applying this character (Figure 2). Webbing of all the material conforms to that of the holotype. Material from the Uluguru mountains (FM 274258–60) is strongly dehydrated and this might have impacted the morphometric measurements. Uluguru material show extreme values for gular flap proportions. Freshly collected material will be necessary to assess the morphometric variation among populations that might potentially recognize one of these populations as being distinct. Given the large amount of molecular difference in Uluguru populations (1.7% mtDNA (ND2) pairwise divergence from joint East Usambara and Nguru populations) the population may be a candidate for a new species.

##### Colour patterning of adults in life

**Figure 6:**
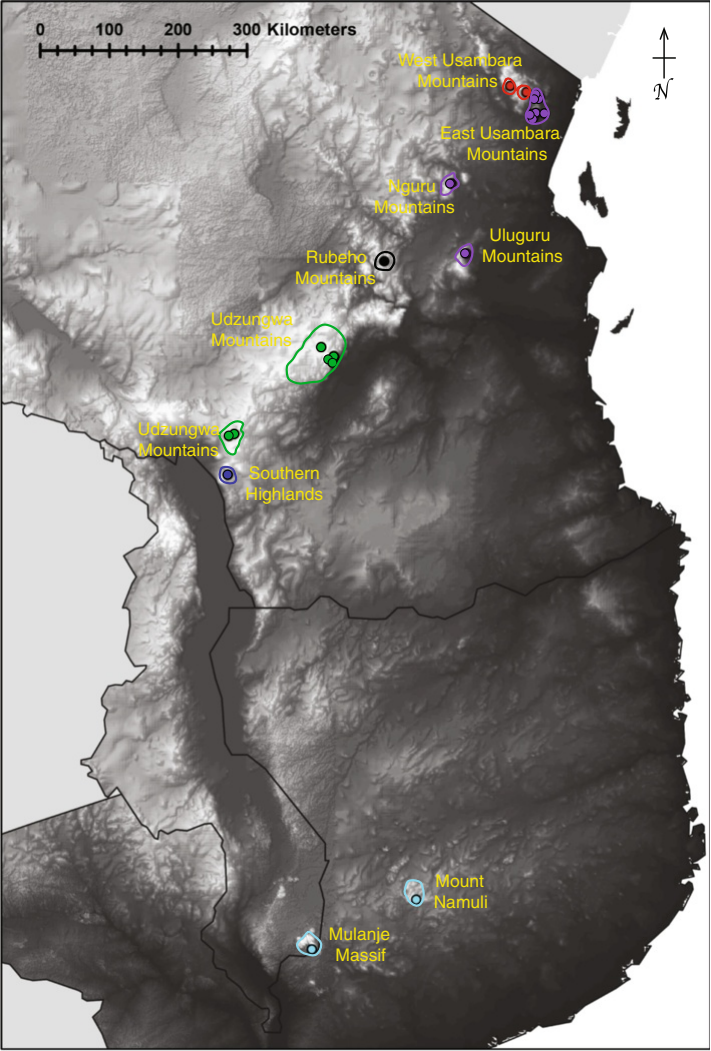
Colour in life of: (top row) *H. burgessi* from Nguru (left male, right female); (second row) *H. davenporti* from Livingstone Mts. (left male, right female); (third row left) *H. ukwiva*; (third row, right) *H. spingularis* from Malawi; (fourth row, left) *H. minutissimus* from Udzungwa Mts.; (fourth row, left) and *H. tanneri* from West Usambara.

See Figure 6 for photo in life. Generally, the females and males resemble the holotype in basic coloration – not showing dichromatic patterns from samples collected. The brown dorsal chromatophores varied in intensity from specimen, and varied within and between population (Nguru and East Usambara for which large series exist) (see Additional file 1: Figure S1 and S2). It should be noted that the Nguru material was prepared differently and much more recently than most of the East Usambara material compared. Some differences might reflect preservation differences. The intensity of chromotophores sometimes resulted in dark brown mottling (particularly in East Usambara material – see Additional file 1: Figure S1). The majority of specimens either had lateral dark edged white stripes (either thin or irregular in size and outline) ending anteriorly in a narrow stripe meeting at the snout or a triangle covering the snout. The ventral side is of a lighter cream coloration.

##### Sexual dimorphism

Females attaining a much larger size than the males (Figure 2). Asperities of the dorsum are less visible in the female and absent from the ventral side in females. Males are easily distinguished from the females during the breeding season by their characteristic rounded, slightly narrowing anteriorly gular sac (Figure 4).

##### Advertisement call

No advertisement call is known but Stevens [3] reported the males making a “weak, rasping high-pitched “tcheek-tcheek” call, believed to serve a territorial function in the close relative *H. spinigularis* occurring in Malawi and Mozambique. Stevens [3] questioned whether this might also be an advertisement call but speculated that the restricted breeding area and season in this species might have “obviated the necessity of a mating call.” Vonesh (in litt.) conducted intensive survey of this species over a two-year period in Amani Nature Reserve and never heard calling males, which is suggestive of a lack of advertisement or territorial calls in *H. burgessi*.

##### Etymology

The species is named for Prof. Neil Burgess, who has made and continues to make enormous efforts towards conserving Tanzania’s forests, which this new species survival depends upon. The species is also restricted to the Eastern Arc Mountain region – an area which Neil has particularly devoted considerable time and energy to understand and preserve.

##### Distribution and conservation

The species is known to occur in East Usambara, Nguru and Uluguru Mountains in Tanzania (Figure 5, Table 4). The species has been collected at high altitudes in, and on the edges of submontane forests. Vonesh [18] (p. 281) commented on the ecology and behavior of this species (then referred to as *H. spinigularis*) saying “it breeds during both annual rainy seasons by attaching its eggs to vegetation overhanging permanent or semi-permanent ponds or swamps. Mean clutch size is 89 eggs. During the long rains of 2002, over 75% of reproductive activity occurred during the first 30 days of the rainy season early March to early April.” Observations of females attaching eggs to vegetation overhanging permanent or semi-permanent ponds or swamps were also made in the Nguru populations of *H. burgessi* (MM, pers. obs.).

**Table 4:** **Species, altitudinal range, habitat and available area of occurrence**

**Species**	**Altitudinal range**	**Habitat**	**Expected area of occurrence**
*H. burgessi*	East Usambara:	Submontane forest	14,774 km^2^
	900–1100 m		
	Nguru: 900–1000 m		
	Uluguru: 980 m		
*H. davenporti*	Livingstone: 2010 m	Montane forest edge	28 km^2^
*H. minutissimus*	Njombe: 2010 m	Montane forest edge and grassland	14,904 km^2^
	Udzungwa:		
	1680–1970 m		
*H. spinigularis*	Malawi: 690 m	Submontane forest and forest edge	5,488 km^2^
	Mozambique: 1250 m		
*H. tanneri*	West Usambara:	Submontane forest and forest edge	4 km^2^
	1310–1650 m		
*H. ukwiva*	Rubeho: 1660 m	Montane forest edge	1,179 km^2^

#### *Hyperolius davenporti* sp. nov.

##### Holotype

**Figure 7:**
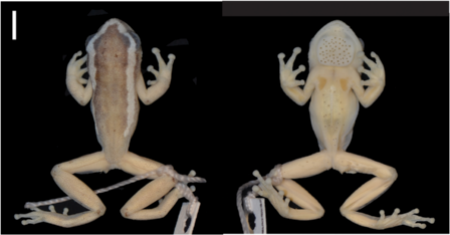
Dorsal and ventral views of the holotype of *H. davenporti* MTSN 7465. Bar = 5 mm.

MTSN 7465 (male) collected in Sakara Nyumo Forest Reserve, Livingstone Mountains, Southern Highlands, Tanzania (−9.8389 S, 34.60781 E, 2010 m) on 14^th^ January 2011 by Michele Menegon (Figure 7).

##### Paratypes

*Females*: MTSN 7453, and MTSN 7464. *Males*: MTSN 7455, MTSN 7456, MTSN 7457–7463, and MTSN 7467. Juvenile: MTSN 7466. Same collection data as holotype.

##### Diagnosis

Horizontal pupil with distinctive gular flap in males. As with most other members of the spiny-throated clade (*H. spinigularis, H. burgessi, H. minutissimus,*), *H. davenporti* also has the presence of dermal asperities (including the body and chin region) on the ventrum, unique amongst *Hyperolius*. The presence of asperites on the gular flap diagnoses this species from *H. tanneri*, for which they are absent. The even distribution of dermal asperities across the gular flap differs from the anteriorly and medially distributed asperities in both *H. minutissimus* and *H. ukviwa.* Furthermore, in males, the species has a distinctive gular flap different in various combinations to other members of the genus. *H. davenporti* males have a rounded gular flap which is not bilobed, distinguishing it from *H. spinigularis* (Figures 2 and 4). Furthermore, the shape of the gular flap in males is different from *H. burgessi* in being wider than the height and shaped more equally in the anterior and posterior ends of the flap (Figures 2 and 7, Tables 1 and 2). Based on molecular comparisons the species is also genetically distinct from close relatives, and is minimally 5.7% pairwise divergent from its closest relative, based on mtDNA (ND2) (Table 3; see Figure 1). *Hyperolius davenporti* has an allopatric distribution with respect to all other species in the complex (Figure 5).

##### Description of holotype

Small to moderate sized hyperoliid. Horizontal pupil. Snout blunt slightly rounded. Canthus rostralis angular, being slightly convex on the horizontal plane and slightly concave on the vertical plane. Distance between eyes is 4.0 mm and the inter orbital distance is 2.4 mm. The inter-narial distance is 1.9 mm, almost subequal to the narial distance to the eye (2.0 mm). The nostril to snout is 1.0 mm. The width of head (7.1 mm) equaling 0.37 length of body (19.1 mm). The gular flap is wider (5.3 mm) by 1.10, than it is in height (4.8 mm). The gular flap is rounded, thickened, and not bilobed. It is marked by black asperites (*ca*. 50) evenly distributed across the whole of the gular flap. Some asperites, sparsely distributed, though more concentrated, on the apex of the chin (mentum). Tibio-tarsal articulation of the adpressed hind limb reaching the eye. Tibio-tarsal (9.3 mm) is almost equal to thigh length (9.0 mm). The tibiable fibulare length is 5.8 mm. The toes have expanded fleshy discs and the foot is 6.2 mm. Webbing is extensive reaching the base of the fleshy discs on all toes apart from the first toe where it only reaches the first tubercle. The forelimb length is 4.6 mm, less than the hand length (5.5 mm). The hands have expanded, rounded fleshy discs. Webbing just reaching distal subarticular tubercle of the outer finger, reaching distal subarticular tubercle of the 4th toe on both sides. Dorsal skin surface granular with a single minute black asperities surmounting many of the granules. Ventral skin surface strongly granular with black asperities restricted to the mentum, gular disc, abdomen and undersurfaces of the femur. Ventral asperities much more prominent than those of the dorsum.

##### Paratypes

Head and body proportions are in close agreement with those of the holotype (Figure 2, Table 1). The distribution of the asperities of the males are in close agreement with that of the holotype. The proportions of the gular flap, diagnostic for the species, show some variation which mean the boundaries of diagnosing this species are in some cases slightly overlapping (Figure 2). Webbing of all the material conforms to that of the holotype. One single specimen (MTSN 7466) had not fully metamorphized and measured 14.3 mm (Snout-Urostyle) with a tail length of 13.8 mm.

##### Colour patterning of adults in life

See Figure 6 for photo in life. Generally the females and males resemble the holotype in basic coloration. The brown dorsal chromatophores varied slightly in intensity amongst specimens (see Additional file 1: Figure S3). The intensity of chromotophores sometimes resulted in dark brown mottling. The majority of specimens either had lateral dark edged white stripes (either thin, broad and regular in shape or irregular in size and outline) ending anteriorly in a narrow stripe meeting at the snout or a triangle covering the snout. The ventral side is of a lighter cream colouration.

##### Sexual dimorphism

Females attaining a much larger size than the males (Figure 2). Asperities of the dorsum weaker in the female and absent from the ventral side in females. Males are easily distinguished from the females during the breeding season by their characteristic rounded and wide gular sac (Figure 4).

##### Advertisement call

Neither advertisement or territorial calls in *H. davenporti* were recorded or heard, however survey time in the area (5 days) was relatively short. Little can be concluded on whether it resembles its congeners *H. burgessi* or *H. spingularis* as no call is known from these species.

##### Etymology

The species is named after Dr. Tim Davenport, who has made substantial contributions towards conserving Tanzania’s forests, in particular the Southern Highlands and Livingstone Mountains of Tanzania. The Livingstone Mountains are the only known locality of this species.

##### Distribution and conservation

The species is only known from Sakara Nyumo Forest Reserve, Livingstone Mountains, Southern Highlands (Figure 5, Table 4). Specimens were collected in shallow ponds on the forest edge in a large swampy area. Species were not found in the open and were always on the forest edge. The area in the Livingstone Mountains comprises a number of fragmented forest patches where the species might also be found. However, based on sampling carried out this was the only location where this species has so far been recorded. Further sampling will be required to establish whether the species is truly restricted to this single forest reserve or more broadly in the Livingstone Mountains or even across the whole Southern Highland region.

#### *Hyperolius ukwiva* sp. nov.

##### Holotype

**Figure 8:**
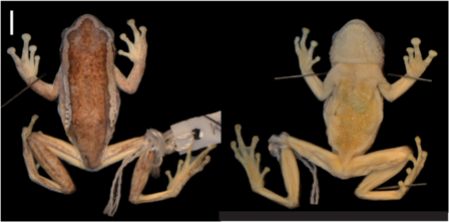
Dorsal and ventral views of the holotype of *H. ukwiva* MTSN 5064. Bar = 5 mm.

MTSN 5064 (KMH 35846) (female) collected in Ukwiva Forest Reserve, Rubeho Mountains, Tanzania (−7.11186 S, 36.64058 E, at 2060 m) on 6^th^ November 2006 by Frontier-Tanzania (see Figure 8).

##### Paratypes

*Male*: KMH 36053 (male), *Female*: MTSN 5085 (KMH 36056). Same collecting locality as holotype but collected on 5^th^ November 2006.

##### Diagnosis

Horizontal pupil with distinctive gular flap in males. As with most other members of the spiny-throated clade (*H. spinigularis, H. burgessi, H. davenporti, H. minutissimus*), *H. ukwiva* also has the presence of dermal asperities (including the body and chin region) on the ventrum, unique amongst members of the genus *Hyperolius*. The presence of asperities on the gular flap diagnoses this species from *H. tanneri*, for which they are absent. The even distribution of dermal asperities on the gular flap differs from the anteriorly positioned distribution of asperities, also found on the chin, in *H. minutissimus* and *H. ukviwa*. Furthermore, in males, the species has a distinctively shaped gular flap, different from *H. minutissimus* in being bilobed and wider than its height (Figures 2 and 4). Females are larger in *H. ukviwa*, reaching sizes >25 mm, substantially larger than females of *H. minutissimus* (18–24 mm) (Figures 2 and 3, Tables 1 and 2). Based on molecular comparisons the species is also genetically distinct from close relatives, and is minimally 7.0% pairwise divergent from its closest relative, based on mtDNA (Table 3; see Figure 1). *Hyperolius ukwiva* has an allopatric distribution with respect to all other species in the complex (Figure 5).

##### Description of holotype

Moderate to large sized hyperoliid. Horizontal pupil. Snout blunt slightly rounded. Canthus rostralis angular, being slightly convex on the horizontal plane and slightly concave on the vertical plane. Distance between eyes is 5.3 mm and the inter orbital distance is 3.1 mm. The inter-narial distance is 2.5 mm, almost subequal to the narial distance to the eye (2.6 mm). The nostril to snout is 1.3 mm. The width of head (9.4 mm) equaling 0.34 length of body (28.0 mm). Gular disc/flap is absent. Tibio-tarsal articulation of the adpressed hind limb reaching the eye. Tibio-tarsal (13.4 mm) is almost equal to thigh length (13.5 mm). The tibiable fibulare length is 8.7 mm. The toes have expanded fleshy discs. Webbing is extensive reaching the base of the fleshy discs on all toes apart from the first toe where it only reaches the first tubercle. The forelimb length is 7.2 mm, less than the hand length (8.4 mm). The hands have expanded, rounded fleshy discs. Webbing just reaching distal subarticular tubercle of the outer finger, reaching distal subarticular tubercle of the 4th toe on both sides. Dorsal skin surface granular with sparsely distributed single minute black asperites surmounting granules. Ventral skin surface strongly granular, particularly on the mid ventral region with large rounded raised surfaces. No asperities present on ventral region.

##### Paratypes

Head and body proportions in close agreement with those of the holotype (see Figure 2; Table 1; Additional file 1). The distribution of the asperities of the single male (see comment below) are medially and anteriorly concentrated on the gular flap (see Figure 4). Webbing of all the material conforms to that of the holotype.

##### Colour patterning of adults in life

See Figure 6 for photo in life. Generally the female and male resemble the holotype in basic coloration. The dorsum is described in field notes as being “brown with two light grey-brown stripes from nose to the hindlegs” for all three specimens. The ventrum is described as sunshine “yellow”. The legs and arms are similarly colored dorsally and ventrally.

##### Sexual dimorphism

Females attaining a much larger size than the males (Figure 2). Asperities of the dorsum weaker in the female and absent from the ventral side in females. Males are easily distinguished from the females during the breeding season by their characteristic gular sac (Figure 4).

##### Advertisement call

No calls were detected or recorded during collection of these three specimens, only one of which was a male.

##### Etymology

The species is named after the forest area (Ukwiva) from where the type series was collected. The specific epithet is considered to be a noun in apposition.

##### Distribution, ecology and conservation

The species is only known from Ukwiva Forest Reserve, in the Rubeho Mountains (Figure 5; Table 4). Specimens were collected in and around the edge of montane forest. Collecting across the Rubeho Mountains, although only relatively recent, has been quite extensive (Rovero, et al. [19] so its localized distribution might not just be a function of restricted sampling.

##### Comment

It should be noted that we would have preferred to describe the holotype as a male (e.g. KMH 36053), in line with the designation of males for all other members of this group of hyperoliids. However, the only known male paratype (KMH 36053) currently cannot be located at UDSM or MTSN, and thus measurements were not possible. Data on this male were taken from photographs and observations made in Tanzania at the time (LL and MM).

#### Further remarks on *Hyperolius minutissimus*

##### Distribution and conservation

Commenting on the distribution and ecology of *Hyperolius spingularis* Schiøtz [20] (p.180) stated “a search for it at the Udzungwas revealed it both in forest and in very open farmland, and it may be found wherever suitable habitats exist in the eastern Tanzanian-Malawi highlands.” According to our records and based on the clustering of Schiøtz’s material with our samples of *H. minutissimus,* we can confirm that these comments refer only to *H. minutissimus,* with *H. spingularis* not recorded from the Udzungwas. Therefore the records given by Schiøtz and Westergaard [4] refer to *H. minutissimus.* On p.167 Schiøtz [20] reports samples collected between Kilosa and Dabaga and these refer to localities reported in Schiøtz and Westergaard [4] and are limited to Udzungwa area – and not beyond, towards Kilosa, that might suggest potential presence in Rubeho of *H. minutissimus*. Currently, we only record the presence of *H. ukwiva* from Rubeho mountain region. *H. minutissimus* is found in forest and grassland habitats in the Udzungwa Mountains and Njombe, the latter in the region of the Southern Highlands. A wider distribution of *H. minutissimus* in the Southern Highlands and beyond into Malawi is as yet unconfirmed and future sampling will be required to establish if it occurs in these areas.

##### Advertisement call

**Figure 9:**
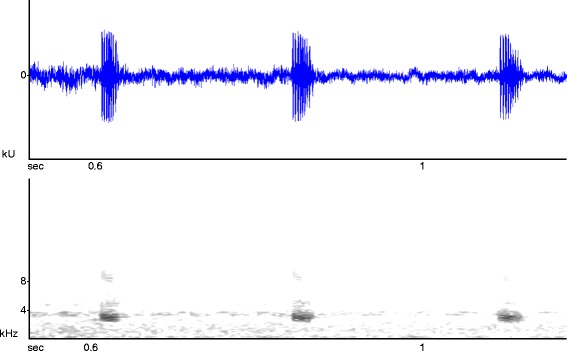
Sonogram of *Hyperolius minutissimus.*

The advertisement call is, as described by Schiøtz, [20], a fast series of quiet, unmelodic clicks, typically repeated about 3 times per second with a dominant frequency of 3.40 and a standard deviation of 0.02 kHz (Figure 9). Calls were recorded both in dense forests and open wetlands. It is interesting to speculate on the potential evolutionary scenarios for differences between spiny-throated species calls. The presence of a call in *H. minutissimus* and apparent absence in congeners (*H. spinigularis, H. burgessi*, and possibly *H. davenporti*) could be linked to habitat differences –important in determining call structure in organisms. *H. minutissimus* occurs in both forest and grassland habitats – with a relatively wide distribution – different compared to the forest restricted and highly localized species *H. spinigularis, H. burgessi,* and *H. davenporti.* It would be interesting to further investigate this pattern and Stevens’ [3] original speculations that absence of a mating call was linked to such factors (e.g. restricted breeding area and season).

### Key to species of the East African spiny throat reed frogs

Here we present a key that should identify adult male specimens of all presently described species. Due to their similarity, identification of females through a dichotomous key is currently not recommended. Geographical distribution (and/or morphometric/molecular analysis) can help in distinguishing between the morphologically similar female species. Caution is required when identifying small or poorly preserved specimens for which the gular flap might be damaged or desiccated:

- 1a Gular flap with black dotted asperities, species not found in West Usambara Mountains.

2

- 1b Gular flap lacking any asperities, species found in West Usambara Mountains.

*H. tanneri*

- 2a Black dotted asperities evenly distributed across the gular flap.

3

- 2b Black dotted asperities distributed on anterior and mid region of the gular flap.

5

- 3a Gular flap bilobed, species present in Malawi and Mozambique.

*H. spingularis*

- 3b Gular flap not bilobed, species present in Tanzania.

4

- 4a Gular flap rounded with posterior and anterior ends more equal. The gular flap is usually either equal or wider than height, species found in Southern Highlands of Tanzania.

*H. davenporti*

- 4b Gular flap narrowly tapering anteriorly and usually equal or greater in height, species found in East Usambara, Nguru, and Uluguru Mountains.

*H. burgessi*

- 5a Gular flap not bilobed and found in Udzungwa Mountains. Females reach a moderate size 18–24 mm.

*H. minutissimus*

- 5b Gular flap bilobed, and found in Rubeho Mountains. Females reach a large size >25 mm.

*H. ukwiva*

## Conclusions

Based on the description of three new species and refinement of the distribution of *Hyperolius minutissimus* and *H. spinigularis* some conclusions can be made on the diversity and distribution of this group of hyperoliids in East Africa. The geographic extent of *H. spinigularis* can now be strictly confined to Malawi and Mozambique – in line with earlier thoughts outlined by Schiøtz [2] who suggested that the northern Tanzanian population might represent a distinct lineage. Our molecular results indicate the Malawi and Mozambique populations of *H. spinigularis* exhibit a sister relationship. However, the morphological similarities between Malawi and Mozambique *H. spinigularis* cannot currently be evaluated as only a single juvenile specimen has been collected from Mt. Namuli in Mozambique [6], and was therefore excluded from morphometric analyses. An adult has been photographed and resembles the colour pattern of the juvenile, including a distinctive heel spot (W. Conradie, pers. comm.). Careful assessment of other characters will be necessary to assess whether the Mozambique population merits species recognition. The strong biogeographic ties between Mt. Mulanje, Mt. Namuli, and other smaller Mozambican massifs have only recently been highlighted by phylogenetic studies of other endemic montane taxa, including dwarf day geckos [21] and pygmy chameleons [22]. It is possible that continued survey work may uncover additional populations of *H. spinigularis* occurring on the smaller massifs of Mozambique, which can then be assessed in the currently presented phylogenetic and morphological framework.

Most species in the spiny-throated group exhibit relatively small distributions, with *H. tanneri, H. davenporti,* and *H. ukwiva* showing distributions restricted to two or less localities. Current estimates of species range areas are particularly narrow and given the limited remaining forest area in these areas and most of the species association with forest (apart from *H. minutissimus*), species are likely to be threatened by increasing habitat change. It will be important with future sampling of these areas to assess if this is truly the full extent of their distributions or if they are indeed wider, as these scenarios have important conservation implications. Future preservation of these endemic species might require specific species-level conservation management approaches. However, the strategies can also account for the many other threatened species in the area – and provide more assemblage or habitat-wide level management approaches.

### Ethics

All specimens were collected according to international guidelines and research permits to collect and export specimens from relevant countries were acquired (see also acknowledgements).

### Zoobank numbers

#### Hyperolius burgessi

urn:lsid:zoobank.org:act:568E4D94-9 F08-417D-81FB-9BF9F230B7BB.

#### Hyperolius davenporti

urn:lsid:zoobank.org:act:61C3C607-1 F44-4CF9-9885-18990DD5337F.

#### Hyperolius ukwiva

urn:lsid:zoobank.org:act:EEF8A317-0C54-44A6-ACA7-93D59259108D.

## Additional files

Additional file 1:
**Supplementary data on species designations, locality information, and photos of phenotype variation within species.**
Additional file 2:
**Morphological measurements for all specimens.**
