# Supplementary material for: Three new species of spiny throated reed frogs (Anura: Hyperoliidae) from evergreen forests of Tanzania
Source: BMC Res Notes. 2015 Apr 25;8:167. doi: 10.1186/s13104-015-1050-y (PMC4409744; doi:10.1186/s13104-015-1050-y)

**BMC Research Notes: Additional file 1**

**Three new species of spiny throated treefrogs (Anura: Hyperoliidae) from Evergreen forests of Tanzania**

SP Loader^1^, LP Lawson^2,3,4^, and DM Portik^5^, M Menegon^6^

^1^ University of Basel, Biogeography Research Group, Department of Environmental Sciences, Basel 4056, Switzerland

^2^ Committee on Evolutionary Biology, University of Chicago, 1025 E. 57^th^ St. Culver Hall 402. Chicago, IL 60637 USA and Field Museum of Natural History, 1400 S. Lake Shore Dr. Chicago IL, 60605 USA

^3^ Field Museum of Natural History, 1400 S. Lake Shore Dr. Chicago, IL 60605 USA

^4^ University of Cincinnati, 614 Rieveschl Hall. Cincinnati, OH 45221, USA

^5^ Museum of Vertebrate Zoology and Department of Integrative Biology, University of California, 3101 Valley Life Sciences Building, Berkeley, California 94720, USA

^6^ Tropical Biodiversity Section, Science Museo of Trento, Via della Scienza e del lavoro, 38122 Trento, Italy.

**Table S1.** Alternative species designations for *BEAST alternative species delimitation scenarios. PS = Path Sampling, SS = Stepping Stone, BF = Bayes Factor

| Species Delimitation | | | Species Groups by Locality | PS | | SS | | PS BF | | SS BF | |  |
| --- | --- | --- | --- | --- | --- | --- | --- | --- | --- | --- | --- | --- |
| All *spinigularis* | | | [EU,NG,UL,LI,MU,NA][UD][RU][WU] | -10836.5 | | -10837.73 | |  | |  | |  |
| Each population | | | [EU][NG][UL][LI][MU][NA][UD][RU][WU] | -10813.27 | | -10813.62 | | 46.46 | | 48.22 | |  |
| All tanzanian *spinigularis* | | | [EU,NG,UL,LI][MU,NA][UD][RU][WU] | -10787.96 | | -10789.17 | | 50.62 | | 48.9 | |  |
| Eastern Arc *spinigularis* | | | [EU,NG,UL][LI][MU,NA][UD][RU][WU] | -10782.59 | | -10783.37 | | 10.74 | | 11.6 | |  |
|  | | |  | |  | |  | |  | |  | |
|  |  |  |  |  |  |  |  |  |  |  |  |  |

EU = East Usambara, NG = Nguru, UL = Uluguru, SH = Southern Highlands, MU = Mulanje, NA = Namuli, UD = Udzungwa, RU = Rubeho, WU = West Usambara, LI = Livingstone, and NA = Namuli.

**Table S2.** Referred material. EU = East Usambara, NG = Nguru, UL = Uluguru, SH = Southern Highlands, MU = Mulanje, UD = Udzungwa, RU = Rubeho, WU = West Usambara, LI = Livingstone, and UKW = Ukwiva. All coordinates are WGS84. Paratypes and holotypes marked in bold with designated holotype underlined.

| **Genus** | **Species** | **Museum No.** | **Sex** | **Latitude** | **Longitude** | **Specific Locality** | **Mountain** | **Country** |
| --- | --- | --- | --- | --- | --- | --- | --- | --- |
| ***Hyperolius*** | ***burgessi*** | **BM 1974.295** | Male | -5.01 S | 38.633 E | Amani | EU | Tanzania |
| ***Hyperolius*** | ***burgessi*** | **BM 1974.296** | Male | -5.01 S | 38.633 E | Amani | EU | Tanzania |
| ***Hyperolius*** | ***burgessi*** | **BM 1974.298** | Male | -5.01 S | 38.633 E | Amani | EU | Tanzania |
| ***Hyperolius*** | ***burgessi*** | **BM 1974.299** | Male | -5.01 S | 38.633 E | Amani | EU | Tanzania |
| ***Hyperolius*** | ***burgessi*** | **BM 1974.300** | Male | -5.01 S | 38.633 E | Amani | EU | Tanzania |
| ***Hyperolius*** | ***burgessi*** | **BM 1974.301** | Male | -5.01 S | 38.633 E | Amani | EU | Tanzania |
| ***Hyperolius*** | ***burgessi*** | **BM 1974.302** | Male | -5.01 S | 38.633 E | Amani | EU | Tanzania |
| ***Hyperolius*** | ***burgessi*** | **BM 1974.303** | Male | -5.01 S | 38.633 E | Amani | EU | Tanzania |
| ***Hyperolius*** | ***burgessi*** | **BM 1974.304** | Male | -5.01 S | 38.633 E | Amani | EU | Tanzania |
| ***Hyperolius*** | ***burgessi*** | **CAS 169258** | Female | -5.167 S | 38.6 E | Kwamkoro Forest Reserve | EU | Tanzania |
| ***Hyperolius*** | ***burgessi*** | **CAS 169259** | Female | -5.167 S | 38.6 E | Kwamkoro Forest Reserve | EU | Tanzania |
| ***Hyperolius*** | ***burgessi*** | **CAS 169260** | Female | -5.167 S | 38.6 E | Kwamkoro Forest Reserve | EU | Tanzania |
| ***Hyperolius*** | ***burgessi*** | **CAS 169261** | Female | -5.167 S | 38.6 E | Kwamkoro Forest Reserve | EU | Tanzania |
| ***Hyperolius*** | ***burgessi*** | **CAS 169262** | Female | -5.167 S | 38.6 E | Kwamkoro Forest Reserve | EU | Tanzania |
| ***Hyperolius*** | ***burgessi*** | **CAS 169945** | Female | -5.01 S | 38.633 E | Amani | EU | Tanzania |
| ***Hyperolius*** | ***burgessi*** | **CAS 169946** | Female | -5.01 S | 38.633 E | Amani | EU | Tanzania |
| ***Hyperolius*** | ***burgessi*** | **CAS 169947** | Female | -5.01 S | 38.633 E | Amani | EU | Tanzania |
| ***Hyperolius*** | ***burgessi*** | **CAS 169976** | Female | -5.01 S | 38.633 E | Amani | EU | Tanzania |
| ***Hyperolius*** | ***burgessi*** | **CAS 169977** | Male | -5.01 S | 38.633 E | Amani | EU | Tanzania |
| ***Hyperolius*** | ***burgessi*** | **CAS 169979** | Male | -5.01 S | 38.633 E | Amani | EU | Tanzania |
| ***Hyperolius*** | ***burgessi*** | **CAS 169980** | Male | -5.01 S | 38.633 E | Amani | EU | Tanzania |
| ***Hyperolius*** | ***burgessi*** | **CAS 169981** | Female | -5.01 S | 38.633 E | Amani | EU | Tanzania |
| ***Hyperolius*** | ***burgessi*** | **CAS 169982** | Male | -5.01 S | 38.633 E | Amani | EU | Tanzania |
| ***Hyperolius*** | ***burgessi*** | **CAS 169983** | Male | -5.01 S | 38.633 E | Amani | EU | Tanzania |
| ***Hyperolius*** | ***burgessi*** | **CAS 169984** | Male | -5.01 S | 38.633 E | Amani | EU | Tanzania |
| ***Hyperolius*** | ***burgessi*** | **CAS 169985** | Male | -5.01 S | 38.633 E | Amani | EU | Tanzania |
| ***Hyperolius*** | ***burgessi*** | **CAS 169987** | Male | -5.01 S | 38.633 E | Amani | EU | Tanzania |
| ***Hyperolius*** | ***burgessi*** | **CAS 169988** | Male | -5.01 S | 38.633 E | Amani | EU | Tanzania |
| ***Hyperolius*** | ***burgessi*** | **CAS 169989** | Male | -5.01 S | 38.633 E | Amani | EU | Tanzania |
| ***Hyperolius*** | ***burgessi*** | **CAS 169990** | Male | -5.01 S | 38.633 E | Amani | EU | Tanzania |
| ***Hyperolius*** | ***burgessi*** | **CAS 169991** | Female | -5.01 S | 38.633 E | Amani | EU | Tanzania |
| ***Hyperolius*** | ***burgessi*** | **CAS 169992** | Male | -5.01 S | 38.633 E | Amani | EU | Tanzania |
| ***Hyperolius*** | ***burgessi*** | **CAS 169993** | Male | -5.01 S | 38.633 E | Amani | EU | Tanzania |
| ***Hyperolius*** | ***burgessi*** | **CAS 169994** | Male | -5.01 S | 38.633 E | Amani | EU | Tanzania |
| ***Hyperolius*** | ***burgessi*** | **CAS 169995** | Female | -5.01 S | 38.633 E | Amani | EU | Tanzania |
| ***Hyperolius*** | ***burgessi*** | **CAS 169996** | Female | -5.01 S | 38.633 E | Amani | EU | Tanzania |
| ***Hyperolius*** | ***burgessi*** | **CAS 169997** | Male | -5.01 S | 38.633 E | Amani | EU | Tanzania |
| ***Hyperolius*** | ***burgessi*** | **CAS 169998** | Male | -5.01 S | 38.633 E | Amani | EU | Tanzania |
| ***Hyperolius*** | ***burgessi*** | **CAS 170000** | Male | -5.01 S | 38.633 E | Amani | EU | Tanzania |
| ***Hyperolius*** | ***burgessi*** | **CAS 170001** | Male | -5.01 S | 38.633 E | Amani | EU | Tanzania |
| ***Hyperolius*** | ***burgessi*** | **CAS 170002** | Male | -5.01 S | 38.633 E | Amani | EU | Tanzania |
| ***Hyperolius*** | ***burgessi*** | **CAS 170003** | Male | -5.01 S | 38.633 E | Amani | EU | Tanzania |
| ***Hyperolius*** | ***burgessi*** | **CAS 170004** | Male | -5.01 S | 38.633 E | Amani | EU | Tanzania |
| ***Hyperolius*** | ***burgessi*** | **CAS 170005** | Male | -5.01 S | 38.633 E | Amani | EU | Tanzania |
| ***Hyperolius*** | ***burgessi*** | **FM 274310** | Male | -5.1133 S | 38.75257 E | Magorotto | EU | Tanzania |
| ***Hyperolius*** | ***burgessi*** | **FM 274311** | Male | -5.1133 S | 38.75257 E | Magorotto | EU | Tanzania |
| ***Hyperolius*** | ***burgessi*** | **FM 274312** | Male | -5.1133 S | 38.75257 E | Magorotto | EU | Tanzania |
| ***Hyperolius*** | ***burgessi*** | **FM 274313** | Female | -5.1133 S | 38.75257 E | Magorotto | EU | Tanzania |
| ***Hyperolius*** | ***burgessi*** | **FM 274314** | Female | -5.1133 S | 38.75257 E | Magorotto | EU | Tanzania |
| ***Hyperolius*** | ***burgessi*** | **FM 274320** | Female | -4.89595 S | 38.64135 E | Nilo | EU | Tanzania |
| ***Hyperolius*** | ***burgessi*** | **FM 274321** | Female | -4.89595 S | 38.64135 E | Nilo | EU | Tanzania |
| ***Hyperolius*** | ***burgessi*** | **FM 274322** | Male | -4.89595 S | 38.64135 E | Nilo | EU | Tanzania |
| ***Hyperolius*** | ***burgessi*** | **FM 274323** | Female | -4.89595 S | 38.64135 E | Nilo | EU | Tanzania |
| ***Hyperolius*** | ***burgessi*** | **FM 274324** | Female | -4.89595 S | 38.64135 E | Nilo | EU | Tanzania |
| *Hyperolius* | *burgessi* | MTSN 8238 | Female | -6.030992 S | 37.525667 E | Pemba | NG | Tanzania |
| *Hyperolius* | *burgessi* | MTSN 8240 | Female | -6.030992 S | 37.525667 E | Pemba | NG | Tanzania |
| *Hyperolius* | *burgessi* | MTSN 8241 | Female | -6.030992 S | 37.525667 E | Pemba | NG | Tanzania |
| *Hyperolius* | *burgessi* | MTSN 8247 | Female | -6.030992 S | 37.525667 E | Pemba | NG | Tanzania |
| *Hyperolius* | *burgessi* | MTSN 8259 | Female | -6.030992 S | 37.525667 E | Pemba | NG | Tanzania |
| *Hyperolius* | *burgessi* | MTSN 8260 | Female | -6.030992 S | 37.525667 E | Pemba | NG | Tanzania |
| *Hyperolius* | *burgessi* | MTSN 8265 | Male | -6.030992 S | 37.525667 E | Pemba | NG | Tanzania |
| *Hyperolius* | *burgessi* | MTSN 8266 | Female | -6.030992 S | 37.525667 E | Pemba | NG | Tanzania |
| *Hyperolius* | *burgessi* | MTSN 8267 | Female | -6.030992 S | 37.525667 E | Pemba | NG | Tanzania |
| *Hyperolius* | *burgessi* | MTSN 8271 | Male | -6.030992 S | 37.525667 E | Pemba | NG | Tanzania |
| *Hyperolius* | *burgessi* | MTSN 8273 | Male | -6.030992 S | 37.525667 E | Pemba | NG | Tanzania |
| *Hyperolius* | *burgessi* | MTSN 8278 | Male | -6.030992 S | 37.525667 E | Pemba | NG | Tanzania |
| *Hyperolius* | *burgessi* | FM 274258 | Female | -6.941436 S | 37.7192 E | Uluguru North | ULU | Tanzania |
| *Hyperolius* | *burgessi* | FM 274259 | Male | -6.941436 S | 37.7192 E | Uluguru North | ULU | Tanzania |
| *Hyperolius* | *burgessi* | FM 274260 | Male | -6.941436 S | 37.7192 E | Uluguru North | ULU | Tanzania |
| ***Hyperolius*** | ***davenporti*** | **MTSN 7453** | Female | -9.8389 S | 34.60781 E | Sakara Nyumo Forest Reserve | LIV | Tanzania |
| ***Hyperolius*** | ***davenporti*** | **MTSN 7455** | Male | -9.8389 S | 34.60781 E | Sakara Nyumo Forest Reserve | LIV | Tanzania |
| ***Hyperolius*** | ***davenporti*** | **MTSN 7456** | Male | -9.8389 S | 34.60781 E | Sakara Nyumo Forest Reserve | LIV | Tanzania |
| ***Hyperolius*** | ***davenporti*** | **MTSN 7457** | Male | -9.8389 S | 34.60781 E | Sakara Nyumo Forest Reserve | LIV | Tanzania |
| ***Hyperolius*** | ***davenporti*** | **MTSN 7458** | Male | -9.8389 S | 34.60781 E | Sakara Nyumo Forest Reserve | LIV | Tanzania |
| ***Hyperolius*** | ***davenporti*** | **MTSN 7459** | Male | -9.8389 S | 34.60781 E | Sakara Nyumo Forest Reserve | LIV | Tanzania |
| ***Hyperolius*** | ***davenporti*** | **MTSN 7460** | Male | -9.8389 S | 34.60781 E | Sakara Nyumo Forest Reserve | LIV | Tanzania |
| ***Hyperolius*** | ***davenporti*** | **MTSN 7461** | Male | -9.8389 S | 34.60781 E | Sakara Nyumo Forest Reserve | LIV | Tanzania |
| ***Hyperolius*** | ***davenporti*** | **MTSN 7462** | Male | -9.8389 S | 34.60781 E | Sakara Nyumo Forest Reserve | LIV | Tanzania |
| ***Hyperolius*** | ***davenporti*** | **MTSN 7463** | Male | -9.8389 S | 34.60781 E | Sakara Nyumo Forest Reserve | LIV | Tanzania |
| ***Hyperolius*** | ***davenporti*** | **MTSN 7464** | Female | -9.8389 S | 34.60781 E | Sakara Nyumo Forest Reserve | LIV | Tanzania |
| ***Hyperolius*** | ***davenporti*** | **MTSN 7465** | Male | -9.8389 S | 34.60781 E | Sakara Nyumo Forest Reserve | LIV | Tanzania |
| ***Hyperolius*** | ***davenporti*** | **MTSN 7466** | juvenile | -9.8389 S | 34.60781 E | Sakara Nyumo Forest Reserve | LIV | Tanzania |
| ***Hyperolius*** | ***davenporti*** | **MTSN 7467** | Male | -9.8389 S | 34.60781 E | Sakara Nyumo Forest Reserve | LIV | Tanzania |
| *Hyperolius* | *minutissimus* | FM 274290 | Male | -8.3295 S | 35.939983 E | Ivalla River | UZ | Tanzania |
| *Hyperolius* | *minutissimus* | MTSN 5415 | Male | -8.373333 S | 35.978611 E | Uzungwa Scarp Forest Reserve | UZ | Tanzania |
| *Hyperolius* | *minutissimus* | MTSN 5416 | Male | -8.373333 S | 35.978611 E | Uzungwa Scarp Forest Reserve | UZ | Tanzania |
| *Hyperolius* | *minutissimus* | MTSN 5417 | Male | -8.373333 S | 35.978611 E | Uzungwa Scarp Forest Reserve | UZ | Tanzania |
| *Hyperolius* | *minutissimus* | MTSN 5418 | Male | -8.373333 S | 35.978611 E | Uzungwa Scarp Forest Reserve | UZ | Tanzania |
| *Hyperolius* | *minutissimus* | MTSN 5419 | Male | -8.373333 S | 35.978611 E | Uzungwa Scarp Forest Reserve | UZ | Tanzania |
| *Hyperolius* | *minutissimus* | MTSN 5420 | Male | -8.373333 S | 35.978611 E | Uzungwa Scarp Forest Reserve | UZ | Tanzania |
| *Hyperolius* | *minutissimus* | MTSN 5421 | Male | -8.373333 S | 35.978611 E | Uzungwa Scarp Forest Reserve | UZ | Tanzania |
| *Hyperolius* | *minutissimus* | MTSN 5422 | Male | -8.373333 S | 35.978611 E | Uzungwa Scarp Forest Reserve | UZ | Tanzania |
| *Hyperolius* | *minutissimus* | MTSN 5423 | Male | -8.373333 S | 35.978611 E | Uzungwa Scarp Forest Reserve | UZ | Tanzania |
| *Hyperolius* | *minutissimus* | MTSN 5424 | Male | -8.373333 S | 35.978611 E | Uzungwa Scarp Forest Reserve | UZ | Tanzania |
| *Hyperolius* | *minutissimus* | MTSN 5425 | Male | -8.373333 S | 35.978611 E | Uzungwa Scarp Forest Reserve | UZ | Tanzania |
| *Hyperolius* | *minutissimus* | MTSN 5426 | Male | -8.373333 S | 35.978611 E | Uzungwa Scarp Forest Reserve | UZ | Tanzania |
| ***Hyperolius*** | ***spinigularis*** | **BM 1975.774** | Male | -16.04831 S | 35.71076 E | Chisambo Estate | MUL | Malawi |
| ***Hyperolius*** | ***spinigularis*** | **BM 1975.775** | Female | -16.04831 S | 35.71076 E | Chisambo Estate | MUL | Malawi |
| *Hyperolius* | *spinigularis* | FM 274893 | Male | -16.04831 S | 35.71076 E | Chisambo Estate | MUL | Malawi |
| *Hyperolius* | *spinigularis* | FM 274894 | Male | -16.04831 S | 35.71076 E | Chisambo Estate | MUL | Malawi |
| *Hyperolius* | *spinigularis* | FM 274943 | Female | -16.04831 S | 35.71076 E | Chisambo Estate | MUL | Malawi |
| *Hyperolius* | *spinigularis* | FM 274944 | Female | -16.04831 S | 35.71076 E | Chisambo Estate | MUL | Malawi |
| *Hyperolius* | *spinigularis* | FM 274945 | Male | -16.04831 S | 35.71076 E | Chisambo Estate | MUL | Malawi |
| *Hyperolius* | *spinigularis* | FM 274946 | Male | -16.04831 S | 35.71076 E | Chisambo Estate | MUL | Malawi |
| *Hyperolius* | *spinigularis* | FM 274947 | Male | -16.04831 S | 35.71076 E | Chisambo Estate | MUL | Malawi |
| *Hyperolius* | *spinigularis* | FM 274948 | Male | -16.04831 S | 35.71076 E | Chisambo Estate | MUL | Malawi |
| *Hyperolius* | *spinigularis* | FM 274949 | Male | -16.04831 S | 35.71076 E | Chisambo Estate | MUL | Malawi |
| *Hyperolius* | *spinigularis* | FM 274950 | Male | -16.04831 S | 35.71076 E | Chisambo Estate | MUL | Malawi |
| *Hyperolius* | *tanneri* | CAS 169265 | Male | -4.8 S | 38.5 E | Mazumbai Forest Reserve | WU | Tanzania |
| *Hyperolius* | *tanneri* | FM 274287 | Female | -4.829116 S | 38.51273 E | Mazumbai Forest Reserve | WU | Tanzania |
| *Hyperolius* | *tanneri* | FM 274288 | Female | -4.829116 S | 38.51273 E | Mazumbai Forest Reserve | WU | Tanzania |
| *Hyperolius* | *tanneri* | FM 274289 | Male | -4.74783 S | 38.29743 E | Shume Magamba forest | WU | Tanzania |
| ***Hyperolius*** | ***ukwiva*** | **MTSN 5064** | Female | -8.373333 S | 36.731285 E | Ukwiva Forest Reserve | UKW | Tanzania |
| ***Hyperolius*** | ***ukwiva*** | **MTSN 5085** | Female | -8.373333 S | 36.731285 E | Ukwiva Forest Reserve | UKW | Tanzania |

**Figures**

**Figure S1.** Colour variation in *H. burgessi* (East Usambara).


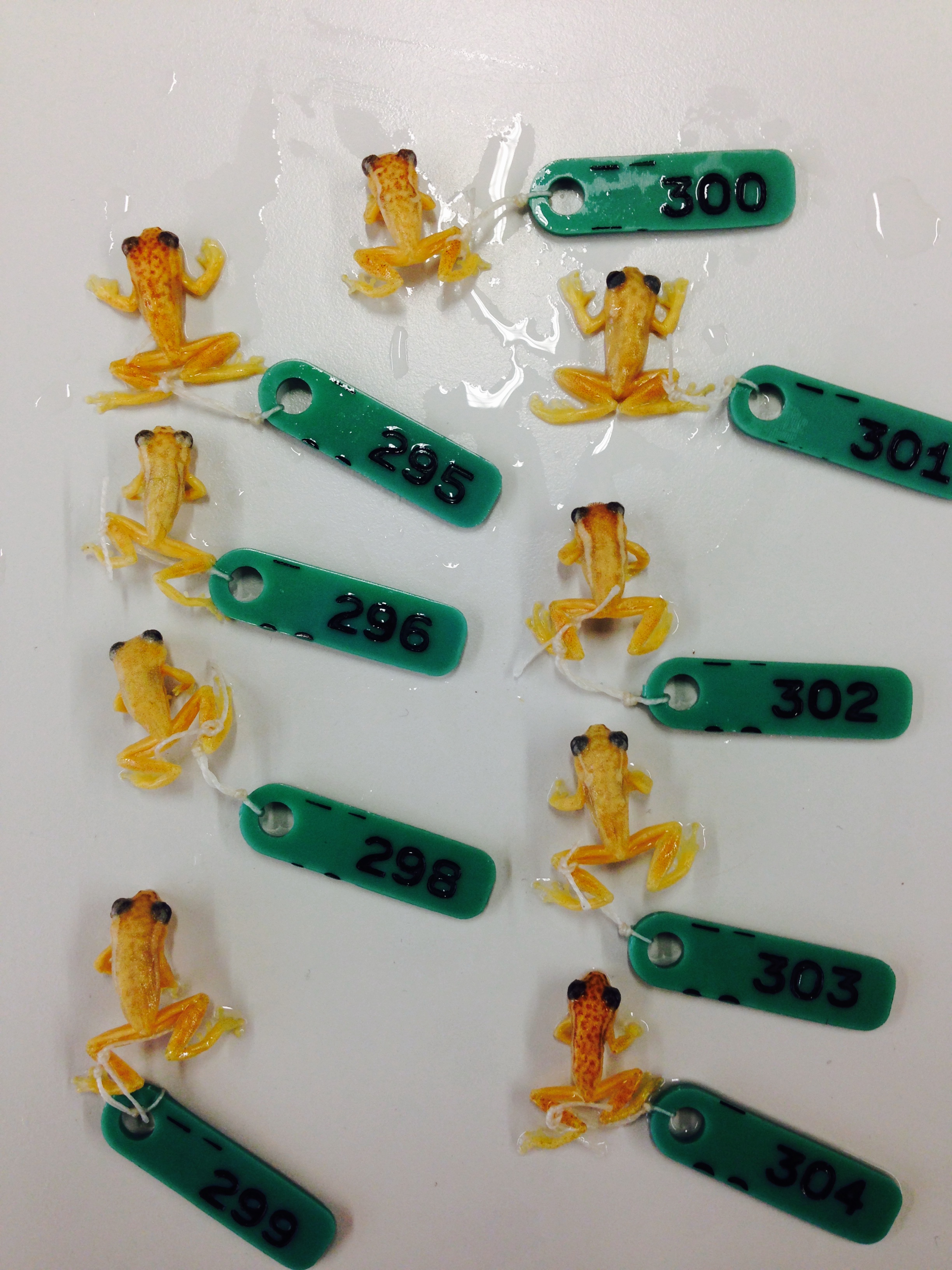


**Figure S2.** Colour variation in *H. burgessi* (Nguru).

**
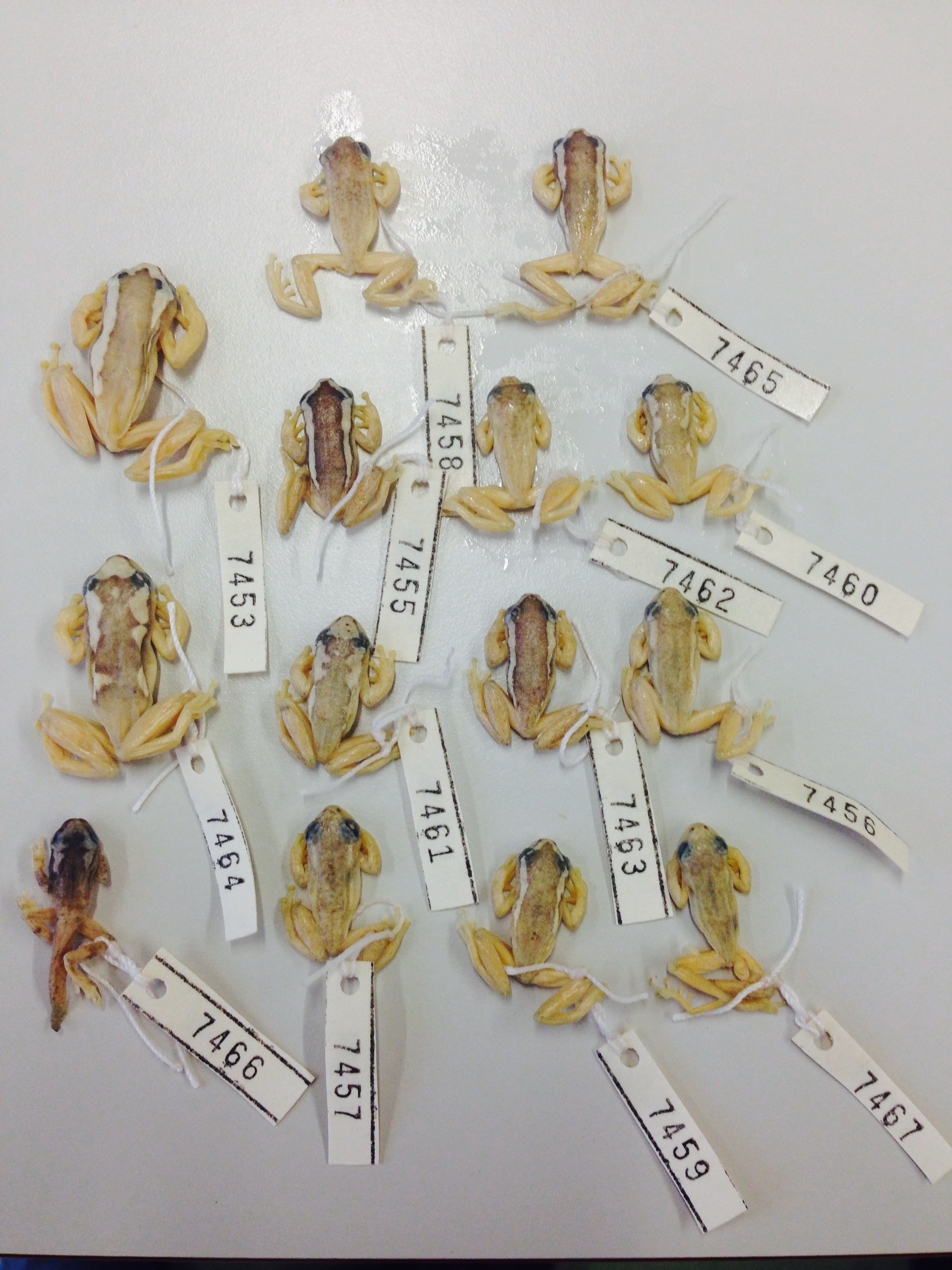
**

**Figure S3.** Colour variation in *H. davenporti* (Livingstone Mountains).


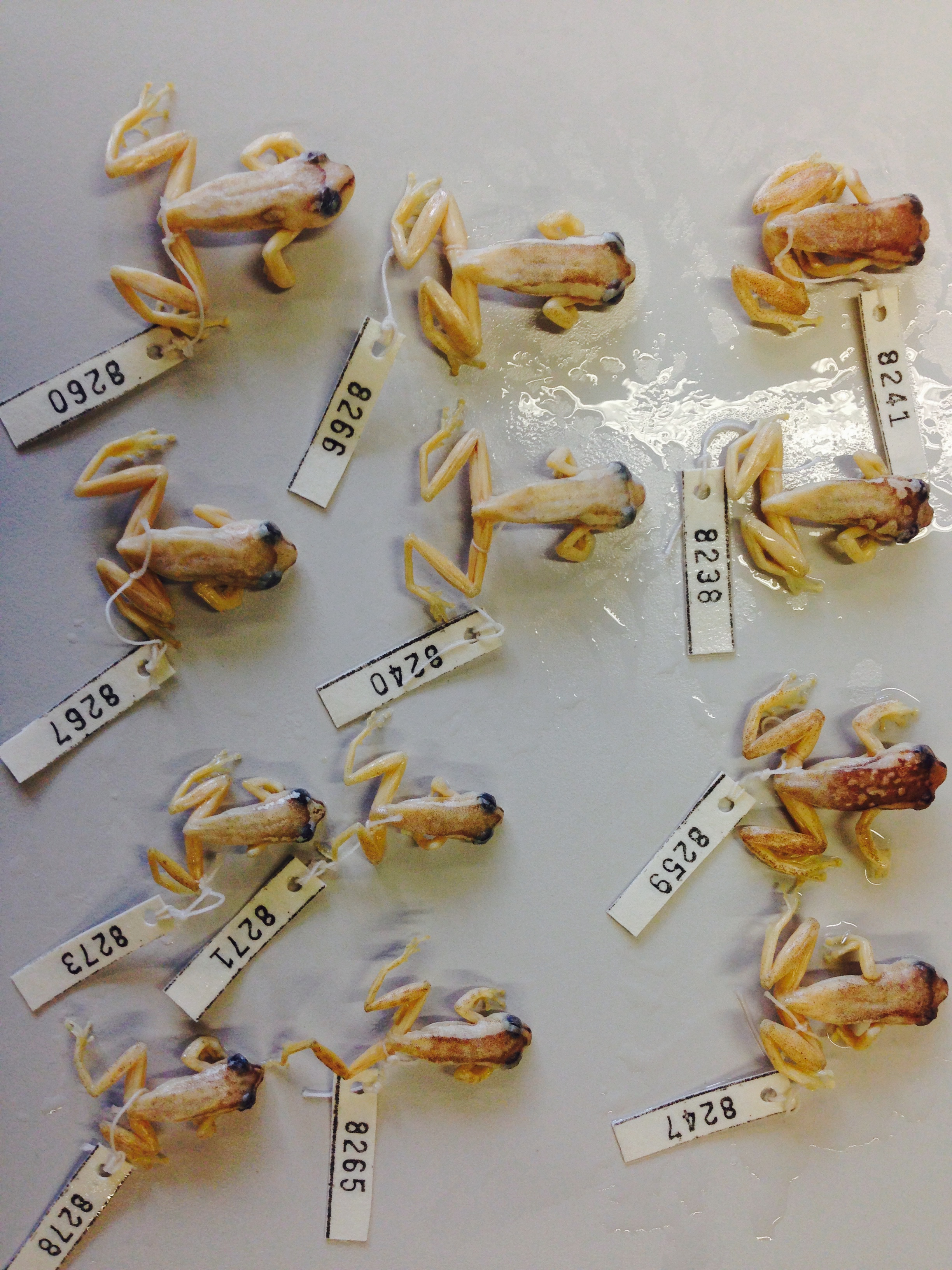

Supplement: Additional file 1: — Supplementary data on species designations, locality information, and photos of phenotype variation within species. [file 13104_2015_1050_MOESM1_ESM.docx]
